# Transnational career advantages: earnings growth of Japanese self-initiated expatriates

**DOI:** 10.3389/fsoc.2025.1646384

**Published:** 2026-01-05

**Authors:** Kenji Ishida

**Affiliations:** Institute of Social Science, University of Tokyo, Tokyo, Japan

**Keywords:** self-initiated expatriates, transnational careers, earnings trajectories, Japanese workers, middle-class migration

## Abstract

**Introduction:**

Japanese workers have experienced prolonged wage stagnation for over 30 years, leading some young and middle-aged people to seek economic opportunities abroad. While self-initiated expatriates (SIEs) represent a growing segment of Japan’s international workforce, their economic outcomes compared to domestic workers remain underexplored. This study examines whether Japanese SIEs achieve superior earnings growth compared to domestic workers and assesses the economic implications of returning to Japan.

**Methods:**

Using longitudinal survey data from the ADIOS-J project (2020-2022) tracking Japanese expatriates and the JLPS dataset for domestic workers, we tested two hypotheses: (1) Japanese SIEs experience higher earnings growth than domestic workers, and (2) returning to Japan economically penalizes SIEs. The analysis controlled for observable and unobservable factors affecting earnings trajectories with random and fixed effects models.

**Results:**

Japanese expatriates experienced approximately 6% annual earnings growth, while domestic Japanese workers’ earnings remained virtually stagnant (around 1% annual growth). Earnings levels varied significantly among expatriate categories—SIEs in multinational companies earned more than domestic workers, while SIEs in Japanese-owned companies had comparable earnings to domestic workers. The apparent economic penalty for SIEs returning to Japan was largely explained by job characteristics and pre-migration conditions rather than the return itself.

**Discussion:**

These findings reveal that transnational careers economically benefit Japanese middle-class workers, challenging conventional views of upward mobility occurring primarily within domestic labor markets. Despite theoretical and empirical issues to be addressed in the future studies, the research contributes to understanding middle-class migration from high-income countries within Asia’s rapidly growing economic centers, suggesting that international mobility can serve as an alternative pathway for career advancement when domestic opportunities are limited.

## 1 Introduction

Japanese workers have suffered from a long wage slump for over 30 years. According to OECD data, Japan’s average annual wage at a constant price, which excludes the effects of price fluctuations, has been around 47,500 US dollars (PPP converted) until 2023 since the 1990s. On the contrary, the OECD average has constantly increased, which means that the wage gap between Japan and other OECD countries has widened. Furthermore, the real wage in South Korea, which is an OECD partner in East Asia, has overtaken Japan’s wage since 2019.

The 30-year-long wage stagnation is a result of the ‘lost decades’ of the Japanese economic market since the early 1990s when the bubble economy burst. During this period, Japanese domestic firms reduced their labor demand while keeping the employment of already existing regular workers. In Japan, where there is a strong linkage between its school-to-work transition system and long-term employment practice (Brinton, 2010), the displacement of new employment affected the opportunity for the labor market entry for young people (Ohta et al., 2008). Accordingly, a considerable number of young Japanese people had to start from non-regular employment, in which both wage level and employment stability were low. The expansion of non-regular employment also gradually yielded middle-aged people who used to have advantages in the Japanese labor market.

During the same period, an increasing number of young and middle-aged Japanese people working overseas was witnessed. A primary group of Japanese workers overseas consists of corporate expatriates (CEs, hereafter), who are dispatched to international branch companies from the headquarters to expand markets and launch local production bases. Meanwhile, against the backdrop of the recession since the 1990s, there has been a gradual number of self-initiated expatriates (SIEs, hereafter) who choose to find their jobs in the destination countries to seek better socioeconomic opportunities and then migrate there for several years or permanently. The wave of migration in the late 1980s and 1990s consisted of Japanese women who graduated from four-year universities and suffered from finding a job in the Japanese labor market (Thang et al., 2006). After that, the SIEs have been prevailing among Japanese young and middle-aged workers with higher education (Kamiya and Niwa, 2018).

According to the official statistics, the number of Japanese workers in private companies in 2017, which is the latest data available, is 463,700, comprising 53.4% of long-term Japanese residents across the world^1^. This figure represents a 1.6 times increase compared to the figure in 1996, 290,178. In particular, East and Southeast Asia are the epicenters of the growing number of Japanese workers, as Japanese companies developed their market opportunities in those regions (Buckley, 2009; Tokunaga and Ikegawa, 2018). Although the number of employees at private companies in each country is not reported, the percentage of Japanese employees at private companies in Asia was 55.5% in 2017, accounting for more than half of the total number of the same group worldwide. This percentage has also increased 1.5 times from 35.7% in 1996.

The present study thus aims to answer the following two research questions with two novel longitudinal survey data for the expatriate and domestic workers: (1) do the Japanese SIEs experience their earnings growth, as one of career outcomes, compared to the domestic workers in Japan, and (2) is returning to Japan penalized or rewarded for the Japanese SIEs’ career?

In what follows, the present study addresses three critical gaps in existing research and advances theoretical understanding of transnational career mobility. First, theoretically, this study challenges the dominance of methodological nationalism in social stratification research by referring to a methodological transnationalism framework. Conventional mobility studies assume that career advancement occurs within bounded national labor markets, overlooking whether transnational experiences can constitute alternative pathways to upward mobility. The present research more explicitly sheds light on this problem. Specifically, it tests whether transnational career experiences yield earnings advantages for Japanese SIEs compared to domestic workers, and whether returning to Japan economically penalizes their careers—questions that directly challenge conventional assumptions about career mobility within bounded national labor markets.

Second, methodologically, this study tries to address a fundamental selection bias problem in return migration research. Previous studies typically compare returnees only with non-migrants, ignoring those who remain abroad—a comparison that conflates return decisions with return outcomes. By utilizing longitudinal data tracking both movers (those who stay abroad and those who return) and stayers (domestic workers), this study better isolates the effects of return migration from pre-existing differences. While acknowledging limitations in sampling design, this approach represents a substantial improvement over cross-sectional comparisons. Third, empirically, by providing systematic evidence on Japanese SIEs’ earnings trajectories, this study reveals that transnational career advantages exist even for migrants from high-income countries in regional labor markets. These contributions advance social stratification research by demonstrating that transnational career mobility represents a viable alternative pathway to economic advancement in an era of domestic wage stagnation in Japan.

## 2 Existing literature and research perspective

### 2.1 Methodological transnationalism

#### 2.1.1 The middle-class positions in methodological nationalism

The present study focuses on the earnings trajectories of the Japanese SIEs as middle-class migrants. In a broad sense, the middle class has been perceived as an intermediary or mixed position [e.g., Erikson and Goldthorpe (1992) and Wright (1997)]. From both Marxist and Weberian perspectives, commonly referred to in social stratification research, social class is initially comprised of three groups in industrial societies: employers (capitalists), workers, and the self-employed (petty bourgeoisie).

The threefold model of the class structure looks insufficient to capture the whole working population in current industrial societies. Under the industrialization process, large-scale businesses give rise to more bureaucratic organizations, creating middle positions to manage front-line workers in the production process efficiently. Furthermore, in postindustrial societies, the middle class has increased in size, comprises the majority of the labor force, and has become heterogeneous. Japan is not an exception. The middle class, comprising managerial, professional, and other nonmanual occupations, accounted for over 50% in 2015, both for men and women, with women being more likely to hold lower nonmanual positions (Ishida, 2022). A more Japan-specific issue is the expansion of so-called non-regular employment since the late 1980s. Unlike regular employment, in which long-term employment and seniority-based salary growth are guaranteed, non-regular employment cannot take advantage of the organizational assets that regular workers are entitled to. This type of duality is pervasive in the Japanese labor market across occupations (Imai and Sato, 2011).

In a theoretical sense, typical social stratification studies, including the abovementioned studies, have relied more or less implicitly on methodological nationalism (Weiss, 2005). In general, methodological nationalism treats societal phenomena from the nation-state perspective in a self-contained way (Wimmer and Glick Schiller, 2002). More specifically, in methodological nationalism, intra- and inter-generational social mobility processes are supposed to occur within one society, independent of others. For example, a commonplace OED (Origin, Education, and Destination) triangle is investigated in line with a domestic social structure and institutional setting. Even for international comparative studies, such as the mobility regime framework [e.g., DiPrete (2002)], what they observe in terms of social stratification processes are the differences among nation-states independent from each other.

#### 2.1.2 Methodological transnationalism in the age of middle-class migration

However, methodological nationalism is now under question as the population size of middle-class migrants is growing. It remains effective in social stratification and inequality studies. Still, it depends on the research topics and questions of interest. Middle-class workers moving between borders, whom I call middle-class migrants in the present study, have yet to be well investigated. As with social stratification research, middle-class migrants are referred to as migrants who occupy middling positions, which are neither globally mobile elites nor lower-skilled labor migrants in transnational migration studies (Conradson and Latham, 2005; Smith, 2005). A straightforward reason why middle-class migrants are understudied is their small population size. As mentioned above, middle-class positions have been occupied by the non-migrant majority in post/industrial societies.

Meanwhile, the situation surrounding the middle class is gradually changing. In contemporary societies, middle-class migrants are emerging (Robertson and Roberts, 2022; Scott, 2019). A broader range of middle-class workers increasingly experience migration in their career trajectories in a globalized economy. Associated professionals and office clerks, who are less regulated by the national certification and licensing systems, are examples of middle-class migrant workers. In terms of size, around 10% of middle-class workers are foreign-born migrants in Western societies (Scott, 2019). While middle-class migrants from emerging economies were initially prevalent in prior research (Erkmen, 2018; Koo, 2016), those from high-income societies are gradually spreading as well (Heisler, 2020; Ho, 2011; Scott, 2006). Accordingly, to understand the middle-class migrants, another theoretical view beyond methodological nationalism appears to be imperative.

Methodological transnationalism is one alternative to address the conceptual issues in situating career mobility accompanied by international mobility (Robinson, 2011; Sklair, 2000; Smith, 2005; Weiss, 2005). In methodological transnationalism, “states (or, more accurately, state agents and agencies) are just one among several factors to be taken into account” (Sklair, 2000: 68). Along with nation-states, any kind of organization, such as both profit and nonprofit organizations and ethnic communities and networks, plays a role in constructing a field of various social interactions across borders (Portes, 2002).

As an important example, transnational labor market research is a pioneer in methodological transnationalism. It specifically focuses on the mobility infrastructure of the transnational labor market, which creates and regulates actual career mobility across borders (Mense-Petermann, 2020; Shire, 2020). Economic activities and competition by profit companies have been global due to the developments and widespread use of technology since the end of the 20th century, such as IT and broader and faster transportation, and then, human mobility across borders for new job opportunities has become frequent as well. However, they have never been spontaneous (Brown, 2000).

In the infrastructure scholarship’s view, they are the outcomes of coordination within the transnational labor market, which various actors deliberately construct through legal, political, social, and cultural processes. In doing so, a specific type of skill or trait is transnationally commodified and priced, which means the transnational labor market is formed. Eventually, aligning with the mobility infrastructure, some migrants become advantaged or disadvantaged compared to other migrants and sometimes even to the majority group in the destination society. As an inspiring example, from the mobility infrastructure perspective, a Japan-centered qualitative study illustrated that Japanese temporary staffing firms (TSFs), which were conceptually intermediary organizations, played an indispensable role in the migration process of Vietnamese IT workers (Muranaka, 2022); Japanese TSFs benefitted not only by coordinating both migrant workers and Japanese firms in their matching processes but also by providing training programs (language, cultural and business etiquettes) for migrant workers to be fitted into Japanese workplaces.

### 2.2 Transnational career mobility of middle-class migrants

The perspective of the mobility infrastructure in the transnational labor market is indeed promising in explaining how transnational job mobility is possible. Meanwhile, there should also be a micro approach to illustrate how migrant workers are actually faced with their transnational career experiences, because only the macro approach may fall into the risk of an over-socialized explanation despite the variation within migrant workers [c.f. Granovetter (1985)]. Apart from the infrastructure approach, the following empirical studies, which cover the middle-class migrant population, investigate migrants’ socioeconomic opportunities through micro-level analyses.

Transnational career mobility is a circular process that involves careers in destinations and upon returning to origin countries (Cassarino, 2004). To understand how these transnational career experiences shape middle-class migrants’ economic outcomes, two interconnected processes require examination. The first—career mobility in destination countries—has been primarily investigated through the lens of immigrant assimilation research. The assimilation thesis, which refers to ‘the decline of an ethnic distinction and its corollary cultural and social differences’ (Alba and Nee, 2003: p.10), postulates that being incorporated into non-immigrant networks and acculturated in the destination society leads to upward economic mobility due to accessing resources exclusive to the majority (Gans, 2007). The second process—career outcomes upon return to origin countries—addresses whether international experience yields returns in origin labor markets, a question particularly salient for migrants from middle- and low-income countries, where skills and networks acquired abroad may command premiums. However, both bodies of research have predominantly focused on migrants moving from lower-income to higher-income countries, leaving the career dynamics of middle-class migrants from high-income countries underspecified both theoretically and empirically. The following sections review these two research traditions and identify critical gaps that motivate the present study.

As the assimilation thesis suggests, US studies utilizing longitudinal earning records show that even the first-generation migrants, who are born outside their destination societies, experience wage growth proportional to the length of stay (Villarreal and Tamborini, 2018, 2024). It appears noteworthy that more educated immigrants enjoy higher wage growth after their arrival (Villarreal and Tamborini, 2018, 2024), which indicates the economic advantage of middle-class migration. Also, a UK study reveals that the first-generation immigrant men’s wage disadvantage is compensated by the length of work experience in the UK (Ochmann, 2024). It also overall supports the positive assimilation hypothesis.

The latter, career mobility upon returning, has been studied primarily in economic literature, particularly for migrants from middle and low-income countries. Because they are thought to accumulate their skills and social capital from the high-income destinations, their experiences and resources obtained through migration are highly valued, which is scarce in their home countries, particularly in the cases of emerging and low-income economies (Dustmann and Weiss, 2007; Hagan and Wassink, 2020). Accordingly, it is hypothesized that returnees earn higher wages than those who have never worked abroad.

By and large, previous studies support that those who returned to their home countries are more highly paid than those without migration experience (non-migrants). For example, in some studies in Ireland, which is a high-income country like Japan, the return migration premium is around 7–10% (Barrett and O’Connell, 2001; Barrett and Goggin, 2010). The positive premium is also reported in the CEE (Central and Eastern Europe) countries and Turkey (Co et al., 2000; Dubu and Rojo, 2021; Martin and Radu, 2012; Tomescu-Dubrow, 2015; Tverdostup and Masso, 2016).

However, regional differences in return migration outcomes warrant careful investigation. Recent East Asian research reveals more complex patterns. Hao et al. (2017) systematically reviewed 143 studies on Chinese returnees, finding mixed evidence: while some successfully leveraged international experience for career advancement, many faced unmet salary expectations and workplace adaptation difficulties. Similarly, Choi and Park (2025) documented how South Korean returnees struggle to convert international credentials into labor market advantages. These contrasting patterns between European and East Asian contexts underscore the importance of regional institutional contexts in shaping transnational career outcomes.

Taken together, two critical gaps emerge from this literature. First, both assimilation and return migration research have predominantly focused on migrants from middle- and low-income countries moving to higher-income destinations. This focus is economically justified: migrants can earn in higher-valued currencies, accumulate valuable skills and networks, and leverage these resources for greater purchasing and bargaining power upon return (Dustmann and Weiss, 2007). However, this logic does not readily apply to middle-class workers from high-income countries, where such resources are already accessible domestically. Regional differences also complicate career opportunities in the transnational mobility process. Second, methodologically, adequately controlling for selection in return migration requires comparing returnees not only with non-migrants but also with those who remain abroad—a comparison rarely achieved in existing studies. Addressing these theoretical and methodological gaps requires longitudinal data that tracks both movers and stayers, allowing for more rigorous examination of how transnational career experiences shape economic outcomes for middle-class migrants from high-income countries.

With respect to the migration from high-income countries, another conceptual framework is the lifestyle migration approach (Benson and O’Reilly, 2009). The lifestyle migration framework underscores that middle-class migrants seek better lives than those in their origin countries, and that socioeconomic achievement is just one of the outcomes of migration. Although career attainment and seeking better lifestyles are not mutually exclusive, middle-class migrants’ income growth is not theoretically guaranteed in the lifestyle migration storyline.

Previous qualitative studies provide some insightful findings; middle-class migrants can retain their economic advantages in the host labor markets while simultaneously seeking their middle-class lifestyle and unique cultural experiences in the host societies (Hof, 2018, 2019). From another angle, a German study found that middle-class migrants from Western countries do not have anxieties of stagnating or downward mobility in Germany. Still, those from other regions do not (Stock, 2024). Those findings imply that middle-class migrants from high-income countries could economically take advantage of their pre-migration experiences. Nevertheless, they have yet to investigate their career trajectories or transnational aspects directly.

In terms of the second issue, the selectivity of return migration, it is essential to follow up on migrants’ transnational mobility to address the selection problem; previous studies have already pointed out that return migration is selective for some individual traits (Constant and Massey, 2002; Wahba, 2015). Technically, longitudinal data that contains cross-border mobility information is the best to address the migrants’ traits in some ways.

To the best of our knowledge, however, most of the previous studies on return migration did not distinguish between migrants who returned to their home countries and those who stayed in their destinations. Some do not directly control for the selection though they are conscious of the issue (Dubu and Rojo, 2021; Tverdostup and Masso, 2016). Several studies have attempted to correct the selection effect upon returning by employing selection models, instrumental variables, propensity score matching, and treatment effect models (Barrett and O’Connell, 2001; Barrett and Goggin, 2010; Co et al., 2000; Martin and Radu, 2012). However, all the datasets consist of all respondents who are already in their home countries. In that data structure, it is possible to adjust the differences between non-migrants and returnees to some extent, but the differences between staying migrants and returnees are not directly controlled. Tomescu-Dubrow (2015) employed random-effect models with a Polish longitudinal dataset, but it also contains only those who are in Poland. A sociological study by Jacobs (2025) is an exception in that the longitudinal data includes information on cross-border mobility of skilled Indian migrants in the US. Still, the outcome is not wage but promotion. Accordingly, considering transnational mobility within individuals is a better way to examine the impact of return migration on career outcomes afterwards.

### 2.3 Transnational career experiences of the Japanese SIEs

#### 2.3.1 The Japanese premium in middle-class migration

The Japanese case offers a critical opportunity to test transnational career mobility theories in a context that differs from existing studies. Unlike the typical pattern of migration from lower-income to higher-income countries, Japanese SIEs represent middle-class workers from a high-income country facing three decades of domestic wage stagnation. This configuration allows us to examine whether transnational mobility can serve as an alternative pathway to economic advancement when domestic career channels are constrained.

In terms of outmigration, a transnational labor market has been opened to the Japanese SIEs by Japanese global companies attempting to develop their business overseas. The labor demand for the Japanese SIEs is a compromise of the Japanese companies to address both localization pressure and keeping Japanese corporate culture.

Since around the 1980s, Japanese companies have expanded their businesses overseas, centered on manufacturing and wholesale trading sectors (Buckley, 2009). The CEs (corporate expatriates) from their headquarters in Japan have primarily managed the local organizations. In return for overseas assignment, they can enjoy a variety of stipends and corporate support, including housekeeping, driving, and their children’s education. In the 1990s, however, when the Japanese economy faced a long-term recession, dispatching the CEs was problematized from a cost-performance viewpoint (Beamish and Inkpen, 1998; Furusawa, 2020). Accordingly, Japanese companies have come to need their localization to reduce their personnel costs.

Meanwhile, Japanese companies have been reluctant to replace managing positions in their local organizations from Japanese to the local staff. As with a German case pointing to perfectionism (Weiss, 2005: 721–2), even Japanese companies overseas require their staff to be like Japanese acculturated workers. As a result, Japanese firms often encounter cultural conflicts (Pudelko and Tenzer, 2011).

There is a supply chain of Japanese companies in the host economic market, and Japanese business customs and culture are a prerequisite within the ecosystem (Ishida et al., 2019). However, few local candidates can sufficiently meet the requirements, primarily due to the Japanese language barrier^2^. Consequently, Japanese-ness has a premium when the considerable size of Japanese supply chains isolates their labor market opportunities exclusively for Japanese candidates, as well as a role of cultural interpreter between the CEs occupying higher positions and local workers, which the Japanese SIEs are expected to play. Concurrently, Japanese people have sought second chances for their careers, particularly since the burst of the bubble economy in the early 1990s. For some of them, working overseas as SIEs appears economically better than remaining in Japan with stagnating career perspectives.

A previous study supports the positive premium; in receiving countries where strong Japanese supply chains exist, the salary level at the same position is higher for Japanese than local staff (Ishida et al., 2019). Meanwhile, in some qualitative studies, they are reported to be precarious and marginal in their workplaces in destination societies (Kamiya and Niwa, 2018; Kato and Kukimoto, 2016; Kawashima, 2010, 2017). According to prior research, Japanese SIEs have to work for Japanese-owned companies at their convenience, and they eventually lack the opportunity to accumulate transnational career experiences that are assumed to be beneficial in migration studies.

#### 2.3.2 International experiences as a trap upon returning to Japan?

When it comes to the SIEs’ return to Japan, the theoretical expectation is more blurred due to a lack of previous studies. Nonetheless, the following aspects in the Japanese labor market imply the pessimistic view of the Japanese returnees from overseas societies.

One is the seniority-based wage growth tied to the long-term employment system in Japanese companies (Yamada and Kawaguchi, 2015). Given the Japanese employment system, it is economically rational for employees to work at the same companies for as long as possible. Put differently, however, the seniority wage system potentially prevents newcomers from the external labor market. Because seniority, that is, the length of working, matters in career attainment in Japan, the transnational experiences of the Japanese SIEs cannot be valued as valid career experiences.

In relation to the prior point, Japanese corporate culture is not well-suited to cultural diversity. One characteristic is the control and cooperation-driven management, which is reported both in management and sociological studies (Ishida and Arita, 2022; Pudelko and Tenzer, 2011). Furthermore, a discourse analysis, based on various articles and reports, also reveals that Japanese companies tend to devalue international experiences (Burgess, 2015).

Previous studies pointed out that the Japanese SIEs were lifestyle migrants who prioritized their private lives more than career advancement (Nagatomo, 2013; Thang et al., 2006). Accordingly, it is plausible to presume that they do not expect so much from their career perspectives (Kato and Kukimoto, 2016). To the best of our knowledge, however, there is no prior study which explicitly focuses on the career mobility of the Japanese SIEs empirically. As well as the previous studies of return migration outside Japan, utilizing longitudinal data is imperative to test the socioeconomic return of returning to Japan as rigorously as possible. In doing so, the present study will contribute to research on transnational career mobility in the case of middle-class migration from high-income countries.

## 3 Limitations in prior research and present hypotheses

The literature reviewed above reveals three critical limitations that constrain its applicability to our research questions about Japanese SIEs’ earnings trajectories and return outcomes.

First, conventional social stratification research relies on methodological nationalism, treating career mobility as processes occurring within bounded national labor markets. This framework cannot address a fundamental question: can transnational career experiences provide economic advantages when domestic labor markets offer limited opportunities? For Japanese middle-class workers facing three decades of wage stagnation, this is not a peripheral question but central to understanding contemporary mobility patterns. The present study addresses this limitation by adopting a methodological transnationalism framework, explicitly comparing earnings trajectories of those pursuing transnational careers with those remaining in Japan.

Second, return migration research faces a substantial selection problem: comparing returnees only with non-migrants conflates selection into return with return effects. If those who return differ systematically from those who stay abroad, simple comparisons produce biased estimates. While some studies employ selection corrections (Barrett and O’Connell, 2001; Martin and Radu, 2012), none compare returnees with those who remain abroad—the most relevant counterfactual. The present study better addresses this problem by tracking both those who stay abroad and those who return, alongside domestic workers, allowing more rigorous examination of return effects.

Third, existing research on Japanese international workers presents contradictory characterizations. Qualitative studies emphasize precariousness (Kamiya and Niwa, 2018; Kawashima, 2010), while quantitative evidence suggests structural advantages (Ishida et al., 2019). This contradiction reflects a deeper limitation: most studies lack longitudinal data or systematic earnings comparisons with domestic workers, making it impossible to assess actual career trajectories. Furthermore, research on return outcomes for Japanese SIEs is virtually nonexistent, despite recent East Asian evidence suggesting that institutional contexts shape return outcomes differently than in European contexts (Hao et al., 2017; Choi and Park, 2025). The present study addresses these gaps by utilizing longitudinal data tracking the earnings trajectories of expatriate workers and comparing them with those of domestic workers.

With the above research gaps in mind, the present study tests the following two hypotheses to answer the research questions raised in the introduction. For the first research question, the corresponding hypothesis is that the growth rates of earnings for the SIEs are higher than those of domestic workers in Japan. Given that their work permits are guaranteed only when local staff cannot take on the job roles, the sponsor companies need to offer the SIE workers higher positions than those of local staff, accompanied by higher wages. Specifically, Japanese companies expect their Japanese SIE workers to intermediate local staff and head managers within their organizations and to be contact persons with other Japanese supply-chain firms in the local society/country because they are presumed to understand the Japanese ways of business customs as Japanese premium (Ishida et al., 2019). On the other hand, non-Japanese-owned or multinational companies do not necessarily require them for SIE workers, who are eventually selected based on their professional skills. Taken together, it can be hypothesized that the Japanese SIE workers are able to obtain higher rates of earnings growth by taking advantage of their market premium, which the Japanese domestic workers do not receive.

Regarding the second research question, the present study hypothesizes that the SIEs’ returning to Japan is a penalty against their earnings. Given the previous studies for Japanese SIEs, the Japanese premium overseas diminishes after returning to Japan for the SIE workers in Japanese-owned companies. Furthermore, given that earnings growth in Japan primarily depends on promotions within organizations due to the typical Japanese employment practice, getting a job through the external labor market does not secure a wage increase. Unlike the CEs, who are embedded into the job rotation system, the SIEs have to find their positions themselves when returning to Japan.

Before proceeding to explain the data used, a note on the setting of the outcome variable should be amended. The present study explicitly focuses on the respondents’ earnings as the outcome of their career mobility for the following two reasons: one is substantial, and the other is for analytical convenience.

For the substantial reason, as mentioned in previous sections, getting better economic opportunities has been a considerable motivation to work in overseas societies for young and middle-aged Japanese workers amid the prolonged stagnation of wage increases since the 1990s in Japan. The growth in earnings can presumably indicate progress in the transnational career mobility across different institutional contexts.

For analytical convenience, the present study is essentially limited to the Japanese middle-class migrants, as shown in the descriptive statistics below. Accordingly, it is hard to investigate their status attainment in a wide range of occupational statuses. Furthermore, employment statuses and job ranks depend on country-specific contexts for their schema and categorizations. In the present study, they are proxy variables measured in accordance with the Japanese labor market situation. Among the career outcomes, therefore, job earning is the best dependent variable to interpret career progress from the transnational career mobility perspective. A greater variety of outcomes should indeed be investigated in future research.

## 4 Data and method

### 4.1 Data and variables

#### 4.1.1 ADIOS-J data

The present study utilizes two kinds of longitudinal survey data to examine the two hypotheses. The first is a three-wave longitudinal dataset obtained through the research project ‘Advancing Dreams in Overseas Societies among Japanese Expatriate Workers (ADIOS-J).’ The first wave of the ADIOS-J survey was conducted online in January 2020, and its target population was Japanese citizens who worked overseas and were less than 50 years old at the time of the survey^3^. The sample was drawn using a non-probability procedure, in which the ADIOS-J project collected respondents through an advertisement on a social media platform (Facebook) and a mailing list of an international staffing agency^4^. The completed sample size of the first wave was 1,011.

The project conducted two follow-up online surveys in January 2021 (the second wave) and February 2022 (the third wave). The survey invitations were sent to 894 of the 1,011 respondents in the first wave, who consented to participate in the follow-up surveys, and 670 and 557 responded to the second and third waves, respectively. Accordingly, the response rates for the second and third waves were 75% (= 670/894) and 62% (= 557/894).

While all respondents in the first wave had paid jobs outside of Japan, some of the respondents became unemployed or jobless in the subsequent waves. Accordingly, the unemployed cases of the total observations are not included in the following analysis because they do not have information on job earnings as the outcome in the present study.

#### 4.1.2 JLPS data

The other data source is the JLPS (Japanese Life Course Panel Surveys) for youth (JLPS-Y) and middle-aged (JLPS-M), which includes workers in Japan during the same survey period from 2020 to 2022 with the ADIOS-J. The JLPS data are analyzed to compare with the ADIOS-J data for earnings trajectories and the associations between earnings and explanatory variables.

The JLPS started in January 2007 as the first wave with the drop-off and pick-up method. Its original target population was comprised of those between 20 and 34 for the JLPS-Y and those between 35 and 40 for JLPS-M who lived in Japan at the time of the survey. The sample was obtained with two-stage random sampling stratified by region, city size, gender, and age group. The follow-up surveys were conducted in January every year, and the additional respondents were amended in 2011 (the fifth wave from 2007) and 2019 (the thirteenth wave from 2007). The former sample was collected by the same birth cohort and sampling method, and the mode of the survey was by mail. The latter consisted of those between 20 and 31 who lived in Japan in 2019 (JLPS-Y refreshment sample, thereafter), and the sampling procedure and survey method were the same as the 2007 sample. In the present study, the datasets in 2020 (the fourteenth wave from 2007), 2021 (the fifteenth wave from 2007), and 2022 (the sixteenth wave from 2007) are used. The number of responses at each wave was 4,891, 4,671, and 4,524 in total. Table 1 represents the response rates for the initial wave for each sample and subsequent waves from 2020 to 2022.

**Table 1:** Response rate for each sample and wave.

	Original sample					Additional sample					Refreshment sample		
		Youth		Middle-aged			Youth		Middle-aged			Youth	
Survey period	Wave	Response	%	Response	%	Wave	Response	%	Response	%	Wave	Response	%
January–April 2007	Wave1	3,367	35%	1,433	40%								
January–March 2011	Wave5	2,232	76%	1,087	85%	Wave1	710	32%	253	31%			
January–March 2019	Wave13	1736	81%	889	88%	Wave9	441	69%	178	74%	Wave1	2,380	36%
January–March 2020	Wave14	1724	82%	871	88%	Wave10	424	68%	175	73%	Wave2	1,697	83%
January–March 2021	Wave15	1700	83%	846	87%	Wave11	409	67%	176	75%	Wave3	1,540	80%
January–March 2022	Wave16	1,682	84%	837	88%	Wave12	401	67%	175	75%	Wave4	1,429	80%

Each response rate is the percentage *p* of responses to the valid survey invitations. Because the second and subsequent waves for each sample are follow-up surveys, high response rates are achieved.

In addition to the ADIOS-J sample, the two kinds of cases are excluded in the following analysis. First, I excluded the tiny number of respondents who lived overseas during the panel due to the use of the JLPS sample in comparison with the ADIOS-J data. Because the number of those cases is five in 2020 and 2021 and two in 2022, their impacts on the data analysis will be negligible. Second, the same with the ADIOS-J data, the unemployed or jobless respondents are excluded from the dataset for each wave.

#### 4.1.3 Variable definitions and summary statistics

Table 2 shows the summary statistics of the outcome and explanatory variables of interest in the present study. The number of observations for each dataset in the table refers to the total observations across the three waves.

**Table 2:** Summary statistics of the variables of interest.

Variables	ADIOS-J		JLPS		Variables	ADIOS-J		JLPS	
	Obs.**	Mean (SD)/%	Obs.**	Mean (SD)/%		Obs.**	Mean (SD)/%	Obs.**	Mean (SD)/%
Natural logged earnings	2053	1.373 (0.685)	7,876	1.198 (0.448)	Employment status	-		10,302	
Living in Japan at survey	2,137	7.58%			Executives		-		2.69%
Age	2,137	37.898 (7.196)	10,302	37.595 (8.410)	Self-employed		-		5.73%
Female	2,106	64.39%	10,302	53.86%	Regular employment		-		66.30%
Educational status obtained in Japan*	2,119		10,251		Non-regular employment		-		25.11%
No education		0.28%		-	Others		-		0.17%
Upper secondary or lower		12.18%		23.79%	Occupational status	2,102		10,110	
Post-secondary		13.36%		30.80%	Clerical		17.65%		26.54%
Undergraduate		56.91%		40.15%	Sales		16.65%		4.05%
Graduate		17.27%		5.26%	Service or retail sales		16.51%		16.29%
Educational status obtained overseas	1996		-		Professional or technical		40.15%		28.84%
No education		66.58%		-	Managerial		8.56%		2.14%
Upper secondary or lower		2.86%		-	Others		0.48%		22.15%
Post-secondary		7.06%		-	Manager positions in large firms	2071	10.09%	10,177	3.74%
Undergraduate		9.12%		-	Monthly working hours	1997	157.078 (60.320)	9,825	170.163 (55.867)
Graduate		14.38%		-	Having spouse	2,103	59.96%	10,295	58.13%
Expatriate status at 2020	2,137		-		Having children	2,108	36.86%	10,238	49.12%
SIE in Japanese companies		26.06%		-	Survey year	2,137		10,302	
SIE in multinational companies		34.96%		-	2020		46.65%		34.49%
Corporate expatriates from JPN		18.02%		-	2021		28.97%		33.15%
Business executives		3.32%		-	2022		24.38%		32.36%
Self-employed or freelance		13.62%		-	Sample type	-		10,302	
Professional or nonprofit organizations		4.02%		-	Original sample since 2007		-		50.78%
					Additional sample since 2011		-		11.79%
					Refresh sample since 2019		-		37.43%

*For the JLPS sample, respondents’ countries of education are not specified. **The number of observations for each dataset is the total observations for the three waves.

The outcome variable is natural-logged annual job earnings at each wave of the ADIOS-J and JLPS datasets. Although its measurement is not detailed, the ADIOS-J surveys used a ten-point scale from one (around one million JPY) to ten (ten million JPY or more) for the respondents’ earnings to make it easier to answer. When asked to answer their job earnings in the ADIOS-J survey, respondents are supposed to answer them in JPY even though they receive their salaries in the currency of their destination societies.

The JLPS surveys ask respondents to indicate the type of wage payment: hourly, daily, weekly, monthly, or annual. Then, they are supposed to answer the detailed values of their wages. The present study calculated their yearly earnings based on their answers and converted them into a ten-point scale, the same as the ADIOS-J version for comparison.

The key explanatory variables are twofold. One is a linear time variable used in both the ADIOS-J and JLPS data, and the other is a dummy variable, whether the respondents lived in Japan at the time of surveys, which is used in the ADIOS-J data. The time variable takes from zero to two, indicating 2020, 2021, and 2022. The living-in-Japan dummy takes one as living in Japan and zero otherwise.

Furthermore, as for the ADIOS-J sample, the respondents’ expatriate status in 2020 (the first wave) is also used as the initial employment status. It comprises the six groups: (1) SIEs in Japanese companies, (2) those in multinational companies (including destination-country-owned companies), (3) CEs from Japanese companies, (4) business executives (regardless of Japanese or non-Japanese companies), (5) self-employed or freelance, and (6) staff in professional or nonprofit organizations. The typical workers in the sixth category are researchers, medical professionals, and officers in international or nonprofit organizations like NGOs.

The regression models also include the following covariates: age, gender (female dummy), education, employment status (for JLPS only), occupation, managerial positions in large firms, monthly working hours, marital status, whether having a child or not and the type of sample (for JLPS only).

The employment status variable is controlled to estimate the growth rate of the outcome variable more rigorously because there are fair differences in the level and growth of job earnings among employment statuses in the Japanese labor market. Specifically, it has five categories: executives, self-employed or freelance, regular employment, non-regular employment, and other non-specified statuses.

For the occupational variable, the ADIOS-J survey asked respondents to choose the following categories that most applied: clerical, sales, service or retail sales, professional or technical, managerial, and other occupations. I particularly distinguished the differences among the sales workers. Because Japanese companies overseas have a sizable labor demand for salespersons (Ishida et al., 2019), store sales workers are closer to service workers. The JLPS survey also asked respondents about their occupations, and I constructed the occupational variable to be comparable with the ADIOS-J dataset.

The variable of whether having managerial positions in large firms is dichotomous. It is a combination of respondents’ job ranks and the size of their firms. For both ADIOS-J and JLPS, the job rank is composed of seven categories: (1) no job title, (2) supervisor, foreman, team leader or equivalent, (3) assistant section chief or equivalent, (4) section chief or equivalent, (5) division chief or equivalent, (6) company president, executive, director, (7) other. Of the seven categories, I defined (4–6) as managerial positions. Also, when working in firms with 300 employees or more, I regarded the respondents as large firm workers. Taken together, if a respondent was in a managerial position in a large firm, the dummy variable took one, and otherwise, zero.

The present study controls respondents’ family situations. Specifically, I use marital status and whether they have children. For the marital status variable, having a spouse is coded as one, and otherwise, it is zero. The child variable is also a dichotomous variable, with one if respondents have children and zero if they do not.

Lastly, for the JLPS sample, I consider the sample types in the following regression models. As mentioned earlier, the JLPS dataset consists of the original, additional, and youth refreshment samples, which started in 2007, 2011, and 2019. Accordingly, I use the dummy variables for the additional and youth refreshment samples to control for the difference in job earnings among the sample types.

### 4.2 Method

#### 4.2.1 Longitudinal regression models

In the present study, I first look at descriptive results of the earnings across the three years among the ADIOS-J and JLPS samples and also between those who were back in Japan after 2020 and those who lived overseas. The descriptive investigation is followed by the multi-variate analyses with the ADIOS-J and JLPS datasets separately. In taking advantage of the longitudinal structure of both datasets, the present study primarily relies on the random effect regression models in the following analysis^5^. I specifically estimate the regression Equation 1.

$$\begin{array}{l} \ln(y_{\mathrm{it}})=b_{0}+b_{1}\mathrm{tim}e_{\mathrm{it}}+b_{2}\text{Japa}n_{\mathrm{it}}+\mathrm{SI}E_{i}b_{3}+b_{4}(\mathrm{tim}e_{\mathrm{it}}\times \mathrm{SI}E_{i})\\ +b_{5}(\text{Japa}n_{\mathrm{it}}\times \mathrm{SI}E_{i})+\mathrm{Zb}+u_{i}+e_{\mathrm{it}} \end{array}$$ (1)

$$u_{i}\sim N(0,\sigma_{u}^{2}),u_{i}⊥x_{\mathrm{it}}$$ (2)

$y_{\mathrm{it}}$ refers to the outcome variable, the ten-scale job earnings at each wave. In the present study, I take its natural logarithmic value. Accordingly, one unit difference in explanatory variables means (exp(x) – 1)% differences in the outcome.

Our key explanatory variables are the linear time variable (time), living in Japan at each wave (Japan), and the expatriate categories in 2020 (SIE). Of these, time and Japan are included only in the ADIOS-J dataset, as explained above. The reference category for SIE is the SIEs in Japanese-owned companies, and each dummy variable for SIE represents other SIE categories. In addition, I take interaction terms between time and SIE and between Japan and SIE. *Zb* is the product of vectors of other covariates and parameters considered in the regression models.

For the first hypothesis that the earnings growth is higher for the SIEs than for domestic workers in Japan, the parameter $b_{1}$ is compared between the ADIOS-J and JLPS datasets, though each parameter is estimated using separate analyses. If the first hypothesis holds, $b_{1}$ is higher in the ADIOS-J dataset than in the JLPS after considering covariates. Also, I check if the growth rates might vary among the SIE statuses by $b_{4}$ .

For the second hypothesis is that the SIE’s earnings after returning to Japan are less than those before returning or staying overseas, I particularly focus on the interaction effects $b_{5}$ . If the hypothesis is supported, the main effect of Japan, which represents the parameter for the SIEs in Japanese-owned firms ( $b_{2}$ ), should be significantly negative. Furthermore, for example, the interaction effect for the CEs should be significantly positive.

I estimate the three random effect models for each sample. The first models include only the explanatory variables of particular interest. The second model adds variables such as gender, age, education, marital status, and children. In addition, the third model counts occupation, managerial positions, and monthly working hours.

In the meantime, as the Equation 2 denotes, the random effect model presumes that the unobserved individual heterogeneity ( $u_{i}$ ) follows a normal distribution with mean zero and a certain variance ( $\sigma_{u}^{2}$ ), and that it is independent of explanatory variables ( $x_{\mathrm{it}}$ ). If these assumptions are not met, the estimated parameters are probably biased. To address this issue, I run fixed effect models for each dataset for a robustness check toward the estimated parameters on which I focus.

#### 4.2.2 Multiple imputations

In performing the aforementioned longitudinal regression models, I may face a considerable reduction in sample size for each dataset. If there were no missing values for all respondents across all waves, 2,137 and 10,302 observations would be available for the ADIOS-J and JLPS datasets, respectively. With complete case analyses, however, sizable observations drop from the analytical datasets. For the ADIOS-J data, the numbers of observations and individuals are 1,763 and 869 with the complete case analysis, resulting in around 17% decreases. Likewise, those are 7,439 and 3,105 for the JLPS data, and around 28% of the potentially available observations are omitted.

The risk of obtaining biased parameters is one of the most crucial concerns for the sample size reduction due to missing values. Ideally, an analytical dataset is assumed to meet the assumptions of missing completely at random (MCAR), which means that missingness for any variable is independent of other variables (Kenward and Carpenter, 2008). Under the situation where the MCAR assumption holds, the complete case analysis theoretically provides unbiased estimates, though their standard errors might be large because of the random reduction of observations. In the meantime, it is almost always rare that the MCAR assumption is satisfied.

To address the sample reduction that potentially results in biased and unstable estimates, the present study employs multiple imputation (MI) techniques. In the MI process, missing values of the variables used are imputed by certain algorithms or regression models with other variables used, as well as auxiliary variables if necessary. After generating multiple complete datasets and estimating parameters with each data, the parameters from all the imputed datasets are combined into the final estimates. In doing so, while utilizing the information of the incomplete dataset as fully as possible, MI can also consider the risk of underestimating standard errors by increasing the sample size because the uncertainty accompanied by the imputation is taken into account in the MI process.

The MI results depend on the sort of imputation methods. In the present study, Amelia II (Honaker et al., 2011) is used for the imputation procedure, and the final estimates are obtained with 50 imputed datasets with all the variables used in the following analyses^6^. I tried changing the number of imputed datasets for the MI process in the following analysis, but the results remain almost the same. Also, to compare the MI results to those in a conventional way, I supplementarily show the results based on the complete case analysis below. As for the complete case analysis, I will also report the results of the Hausman test to check whether the random effect model can tentatively ignore the unobserved heterogeneity, which is controlled with the fixed effect model.

## 5 Results

### 5.1 Descriptive results

**Figure 1:**
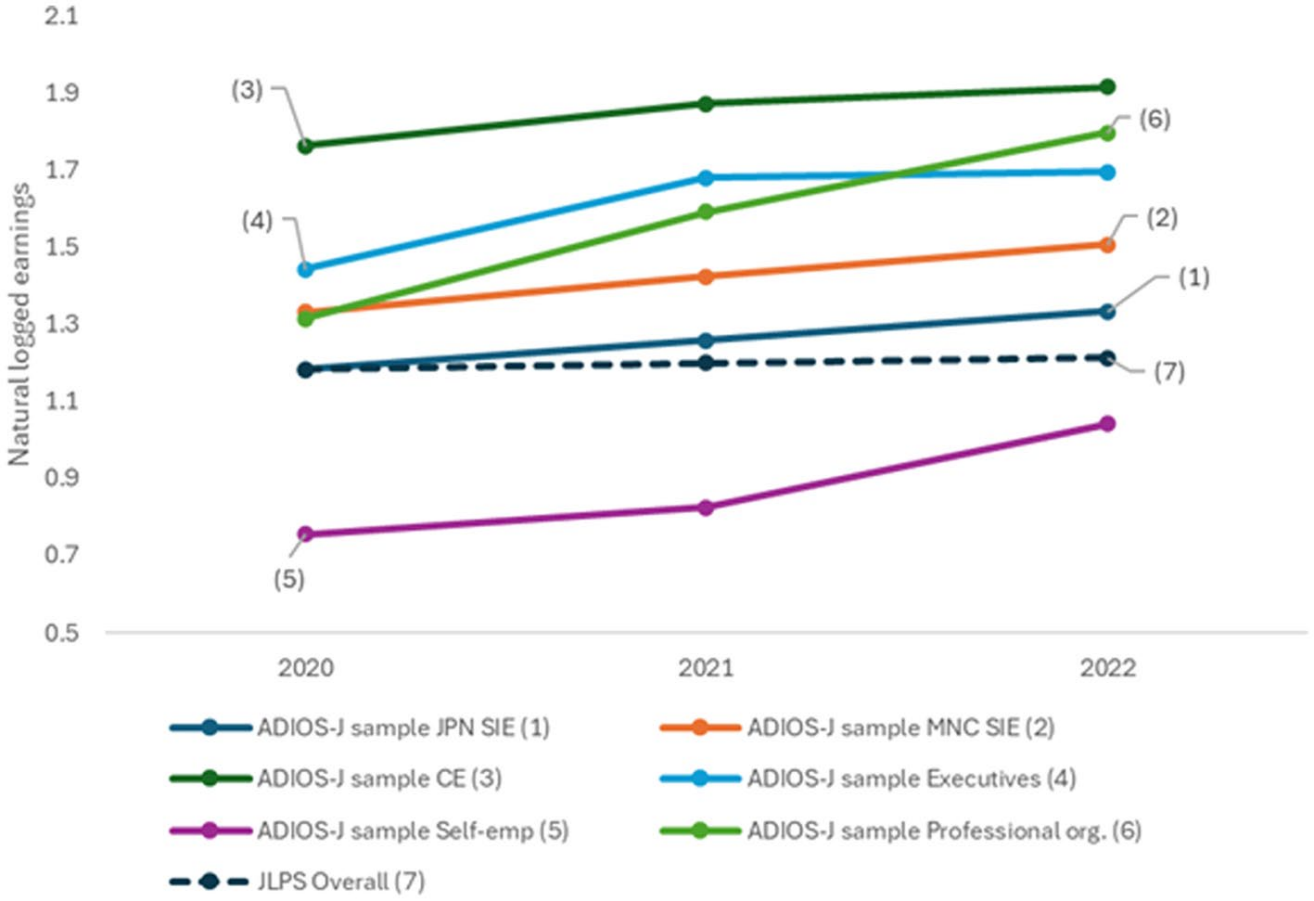
Job earnings trajectories for the ADIOS-J and JLPS samples.

Figure 1 illustrates the trajectories of mean natural logged earnings between the ADIOS-J and JLPS samples. The former is divided into seven groups based on the expatriate statuses at the first wave in 2020. In the ADIOS-J sample, SIEs in Japanese-owned companies (1) had the lowest earnings among employee categories. The relative economic disadvantage of this group aligns with findings from prior research on the Japanese SIE workers overseas. The JLPS sample’s earnings, which are represented as the dashed line, are similar to those of the SIEs in Japanese-owned companies, which are around 1.2 for three years. Accordingly, regarding wage levels, SIE workers in Japanese companies are in a similar economic standing to Japanese domestic workers.

With regard to the first hypothesis, all ADIOS-J groups experienced earnings growth across survey waves. Employee groups (SIEs in Japanese and multinational companies, and CEs) showed similar growth rates of approximately 8% per year (0.075–0.080 points), regardless of expatriate status. This consistent growth pattern among expatriate workers contrasts sharply with the domestic labor market, as discussed below.

The earning trajectory of the JLPS sample was almost flat across the survey waves. On the three-year average, Japanese domestic workers just received a 1% increase per year. Taken together, the descriptive results support the first hypothesis; the earnings growth rate is higher among Japanese expatriate workers, including SIEs in Japanese-owned firms, compared to Japanese domestic workers.

**Figure 2:**
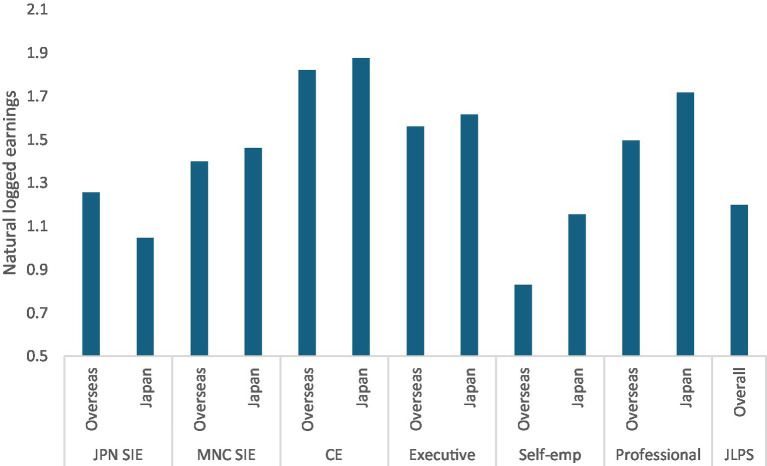
Mean earnings by whether to be in Japan during the survey.

Figure 2 presents mean logged earnings by sample type, expatriate status, and location (Japan vs. overseas). The pattern reveals substantial heterogeneity in return migration outcomes across expatriate categories.

SIEs in Japanese-owned firms experienced an 18.9% earnings decline upon return to Japan (point difference: −0.21). Notably, their earnings in Japan fell below those of domestic JLPS workers, despite earning slightly more while overseas. This pattern suggests a loss of transnational advantages for this group.

In contrast, other expatriate categories showed earnings increases after returning to Japan. SIEs in multinational companies, CEs, and executives gained 5–6%, while self-employed workers and professionals in nonprofit organizations showed substantially higher increases (38.3 and 24.7%, respectively). These divergent patterns suggest that return outcomes depend critically on expatriate status and organizational context, challenging the assumption of a uniform return penalty as predicted by the second hypothesis.

These descriptive patterns provide preliminary support for Hypothesis 1: expatriate workers experienced substantially higher earnings growth than domestic workers from 2020 to 2022. However, evidence for Hypothesis 2 is mixed. SIEs in Japanese-owned firms showed lower earnings after return, falling below domestic worker levels, while other expatriate categories experienced an earnings increase. These divergent patterns suggest that return outcomes vary systematically by expatriate status, warranting multivariate analysis.

### 5.2 Results of random effect models

The descriptive patterns observed above provide initial insights but do not account for respondents’ observable and unobservable characteristics. To rigorously test our hypotheses while controlling for potential confounders, we employ random effects panel regression models (specified in Section 4.2.1). Tables 3, 4 present results for the ADIOS-J and JLPS samples, respectively.

**Table 3:** Random and fixed effect models for the ADIOS-J sample.

	Model 1–1 (RE)		Model 1–2 (RE)		Model 1–3 (RE)		Model 1–4 (FE)	
	Coef	SE	Coef	SE	Coef	SE	Coef	SE
Intercept	1.178***	0.038	0.499	0.298	0.110	0.271		
Japan at survey	−0.134+	0.073	−0.113	0.072	−0.055	0.070	−0.028	0.075
Time (2020 = 0)	0.080***	0.020	0.059**	0.020	0.063***	0.019	0.080***	0.020
Expatriate category at 2020 (ref: JPN SIE)								
MNC SIE	0.144**	0.051	0.072	0.047	0.118**	0.044		
CE	0.594***	0.059	0.451***	0.056	0.405***	0.051		
Executive	0.281*	0.111	0.102	0.104	0.079	0.095		
Self-employed	−0.434***	0.066	−0.474***	0.061	−0.295***	0.057		
Professional organization	0.147	0.104	−0.124	0.099	−0.084	0.091		
Female			−0.300***	0.038	−0.179***	0.036		
Age at survey			0.016***	0.003	0.015***	0.002		
Education in Japan (ref: no education)								
Secondary			0.203	0.285	0.036	0.256		
Post-secondary			0.122	0.286	−0.025	0.258		
Undergraduate			0.321	0.284	0.133	0.256		
Graduate			0.507+	0.287	0.318	0.259		
Education overseas (ref: no education)								
Secondary			−0.304**	0.098	−0.260**	0.089		
Post-secondary			−0.028	0.068	0.051	0.063		
Undergraduate			0.115+	0.063	0.096+	0.057		
Graduate			0.255***	0.054	0.251***	0.049		
Having a spouse			0.019	0.034	0.032	0.031	0.038	0.048
Having children			0.012	0.033	0.034	0.031	0.014	0.046
Occupation at survey (ref: Clerical work)								
Sales					0.056	0.038	0.062	0.048
Service and retail sales					−0.132***	0.039	−0.038	0.054
Professional and technical					−0.023	0.033	−0.027	0.041
Managerial					0.065	0.045	0.027	0.055
Other occupations					−0.324*	0.145	−0.348*	0.164
Manager in large firms					0.205***	0.039	0.107*	0.048
Monthly working hours					0.0031***	0.0002	0.0029***	0.0003
Interaction: Japan × Expatriate Status								
Japan × MNC SIE	0.214*	0.102	0.205*	0.101	0.094	0.097	0.108	0.103
Japan × CE	0.082	0.099	0.061	0.098	−0.045	0.094	−0.074	0.101
Japan × Executive	0.299	0.238	0.259	0.234	0.404+	0.227	0.370	0.242
Japan × Self-employed	0.371*	0.155	0.357*	0.152	0.202	0.149	0.188	0.160
Japan × Professional organization	0.374	0.242	0.372	0.237	0.184	0.226	0.186	0.248
Interaction: Time × Expatriate Status								
Time × MNC SIE	−0.010	0.026	−0.007	0.025	−0.014	0.024	−0.019	0.025
Time × CE	−0.029	0.034	−0.032	0.033	−0.043	0.032	−0.040	0.033
Time × Executive	0.012	0.061	0.015	0.060	0.023	0.058	0.009	0.061
Time × Self-employed	0.003	0.035	0.001	0.034	−0.007	0.033	−0.006	0.034
Time × Professional organization	0.141**	0.052	0.140**	0.052	0.133**	0.051	0.142**	0.052
Rho	0.753		0.707		0.675		0.886	
Obs.	2,137		2,137		2,137		2,137	
Individuals	997		997		997		997	

***p < 0.001, **p < 0.01, *p < 0.05, + *p* < 0.1 (two-tailed test). The number of imputed datasets is 50.

**Table 4:** Random and fixed effect models for the JLPS sample.

	Model 2–1 (RE)		Model 2–2 (RE)		Model 2–3 (RE)		Model 2–4 (FE)	
	Coef	SE	Coef	SE	Coef	SE	Coef	SE
Intercept	1.479***	0.030	0.895***	0.061	0.668***	0.061		
Time (2020 = 0)	0.012***	0.004	−0.0012	0.004	0.001	0.004	0.015***	0.004
Employment status at survey (ref: Executives)								
Self-employed	−0.393***	0.034	−0.347***	0.032	−0.328***	0.031	−0.075	0.047
Regular employment	−0.195***	0.030	−0.164***	0.027	−0.197***	0.027	−0.046	0.044
Non-regular employment	−0.685***	0.033	−0.569***	0.030	−0.491***	0.030	−0.223***	0.048
Others	−0.882***	0.125	−0.828***	0.122	−0.722***	0.123	−0.389**	0.140
Female			−0.286***	0.011	−0.236***	0.011		
Age at survey			0.012***	0.001	0.011***	0.001		
Education (ref: Secondary)								
Post-secondary			0.040**	0.015	−0.003	0.014		
Undergraduate			0.199***	0.014	0.142***	0.015		
Graduate			0.319***	0.026	0.219***	0.025		
Having a spouse			0.080***	0.014	0.083***	0.013	0.017	0.025
Having children			0.007	0.013	0.011	0.013	−0.0037	0.026
Occupation at survey (ref: Clerical work)								
Sales					0.043+	0.023	0.005	0.040
Service and retail sales					−0.069***	0.016	0.006	0.033
Professional and technical					0.108***	0.013	0.046	0.032
Managerial					0.178***	0.031	0.058	0.046
Other occupations					−0.025	0.015	0.015	0.032
Manager in large firms					0.216***	0.023	0.067*	0.032
Monthly working hours					0.0016***	0.0001	0.0010***	0.0001
Sample type (ref: original)								
Additional sample since 2011	−0.041*	0.020	−0.002	0.018	0.009	0.016		
Refresh sample since 2019	−0.229***	0.013	−0.013	0.022	−0.020	0.020		
Rho	0.679		0.602		0.546		0.836	
Obs.	10,302		10,302		10,302		10,302	
Individuals	3,971		3,971		3,971		3,971	

***p < 0.001, **p < 0.01, *p < 0.05, + *p* < 0.1 (two-tailed test). The number of imputed datasets is 50.

Model 1–1 (Table 3) serves as the baseline for the ADIOS-J sample, including key variables of interest. The time variable shows significant positive earnings growth of approximately 8% per year (coef. = 0.080, *p* < 0.001), with no significant variation across employed expatriate categories.

The living-in-Japan coefficient is negative and marginally significant (−0.134, *p* < 0.10), indicating a 12.5% earnings decline upon return for SIEs in Japanese-owned companies. However, significant positive interactions for SIEs in multinational companies and self-employed workers reveal substantial heterogeneity: the former group experienced an 8.3% increase upon return. This divergence suggests fundamentally different return outcomes across expatriate categories.

Consistent with Figure 1, Model 1–1 confirms significant earnings differentials by expatriate status: SIEs in multinational companies earned 15.5% more, and CEs earned 81.1% more, than SIEs in Japanese-owned companies (both *p* < 0.01).

Model 1–2 adds demographic and family variables. The time coefficient remains significantly positive (0.059, indicating 6% annual growth), and interaction patterns remain consistent with Model 1–1.

The living-in-Japan coefficient remains negative and marginally significant (−0.113, *p* < 0.10, implying a 10.7% decline), with interaction patterns similar to Model 1–1. After controlling for demographic variables, the earnings premiums for SIEs in multinational companies and business executives become statistically insignificant, with coefficients declining by approximately 50%. This suggests that much of the earnings differential observed in Model 1–1 reflects compositional differences in age, gender, and education rather than expatriate status per se.

Model 1–3 adds job characteristics (occupation, managerial position, working hours). The time coefficient remains positive and significant (0.063, approximately 7% annual growth), with stable interaction patterns.

Critically, the living-in-Japan coefficient becomes statistically insignificant and declines by 51.3% in magnitude (from −0.113 to −0.055). Similarly, most interaction effects lose statistical significance and decline substantially. These patterns indicate that the apparent return penalty and its heterogeneity across expatriate categories are largely explained by job characteristics rather than return migration per se. This finding challenges the notion of an inherent return penalty for Japanese SIEs.

Table 4 presents parallel models for the JLPS sample (domestic Japanese workers). Given the negligible number of overseas residents in JLPS, these cases were excluded, and return migration variables are not examined.

Model 2–1 (Table 4) includes time, employment status, and sample type. Domestic workers experienced modest but significant earnings growth of 1.2% annually (coef. = 0.012, *p* < 0.001)—substantially lower than the 8% growth observed among expatriates. In Model 2–2, after adding demographic and family variables, the time coefficient becomes insignificant and approaches zero, while the age coefficient becomes significantly positive (0.011). This contrasts with the ADIOS-J sample (Table 3), where both time and age remain significant. The pattern suggests that for domestic workers, earnings growth is driven primarily by seniority (age) rather than labor market experience—consistent with Japan’s seniority-based wage system discussed in Section 2.3.2.

Taken together, these multivariate results provide strong support for Hypothesis 1: Japanese expatriates experienced significantly higher earnings growth (6–8% annually) compared to domestic workers (approximately 1% or less). However, Hypothesis 2 receives limited support: while SIEs in Japanese-owned companies showed lower earnings upon return in baseline models, this apparent penalty was largely explained by job characteristics and pre-migration conditions rather than return migration itself.

### 5.3 Supplementary analyses

Two robustness checks address potential concerns: (1) unobserved individual heterogeneity, examined through fixed effects models, and (2) sensitivity to the multiple imputation procedure, examined through complete case analysis.

Fixed effects models (Models 1–4 and 2–4, Tables 3, 4) control for unobserved time-invariant individual heterogeneity while retaining the multiple imputation approach. For the ADIOS-J sample (Model 1–4), the time coefficient remains significantly positive (0.080, *p* < 0.001), confirming robust earnings growth for expatriates even after accounting for unobserved individual traits. The living-in-Japan coefficient becomes smaller and statistically insignificant (−0.028), with no significant interactions. These results closely parallel Model 1–3, suggesting that unobserved heterogeneity does not substantially alter the key findings.

For the JLPS sample (Model 2–4), the time coefficient becomes significantly positive (0.015, *p* < 0.001, implying 1.5% annual growth)—differing from Model 2–3 where it was insignificant. This occurs because the fixed effects specification cannot separately estimate age effects given the short panel length (three waves), resulting in potential confounding between time and age. Nevertheless, even this upper-bound estimate (1.5%) remains substantially lower than the 8% growth observed among expatriates.

The Hausman test is conventionally used to evaluate whether random effects (RE) or fixed effects (FE) models provide consistent estimates by testing whether coefficients differ significantly between the two specifications. If the null hypothesis of equal coefficients is rejected, FE models are preferred as they better control for unobserved heterogeneity. However, post-hoc testing after multiple imputation remains methodologically underdeveloped for panel data (Grund et al., 2023). We therefore conducted Hausman tests using complete case analysis (Table 5).

**Table 5:** Random and fixed effects models with the complete cases.

	ADIOS-J				JLPS			
	Random effect		Fixed effect		Random effect		Fixed effect	
	Coef	SE	Coef	SE	Coef	SE	Coef	SE
Japan at survey	−0.134+	0.075	−0.136+	0.078				
Time (2020 = 0)	0.077***	0.018	0.088***	0.018	0.003	0.003	0.018***	0.002
Interaction: Japan × Expatriate Status								
Japan × MNC SIE	0.237*	0.100	0.265*	0.105				
Japan × CE	0.040	0.095	0.062	0.100				
Japan × Executive	0.518**	0.192	0.504*	0.199				
Japan × Self-employed	0.331*	0.140	0.331*	0.149				
Japan × Professional organization	0.320	0.203	0.393+	0.216				
Interaction: Time × Expatriate Status								
Time × MNC SIE	−0.026	0.023	−0.026	0.023				
Time × CE	−0.066*	0.029	−0.058*	0.030				
Time × Executive	0.116*	0.058	0.124*	0.060				
Time × Self-employed	0.004	0.029	0.016	0.030				
Time × Professional organization	0.070	0.046	0.060	0.047				
Other covariates	Controlled		Controlled		Controlled		Controlled	
Rho	0.763		0.924		0.765		0.925	
Obs.	1763		1763		7,439		7,439	
Individuals	869		869		3,105		3,105	
Hausman test (RE vs. FE)								
Chi-squared	24.937				436.37			
*df*	21				14			
*p*	0.250				<0.001			

***p < 0.001, **p < 0.01, *p < 0.05, + *p* < 0.1 (two-tailed test). The other covariates are the same as Model 1–3 and 2–3 for the random effect models and Model 1–4 and 2–4 for the fixed effect models in the ADIOS-J and JLPS samples.

Table 5 presents random and fixed effects models using complete case analysis (no missing values on any variables). For the ADIOS-J sample, time coefficients remain significantly positive in both RE (0.077) and FE (0.088) models. The living-in-Japan coefficient is larger and marginally significant in complete case analysis (−0.134 to −0.136) compared to MI results, likely reflecting overestimation due to listwise deletion bias under MAR assumptions. For the JLPS sample, results are nearly identical between MI and complete case approaches.

The Hausman test does not reject the null hypothesis for the ADIOS-J sample, supporting the validity of the RE specification. In contrast, the null is rejected for the JLPS sample, favoring the FE model. Importantly, even using the FE estimate for JLPS (1.5% annual growth), domestic workers’ earnings growth remains substantially lower than that of expatriates (6–8% annually). These robustness checks consistently support Hypothesis 1 regarding higher earnings growth for expatriates, while providing limited support for Hypothesis 2 regarding return penalties.

## 6 Discussion

This study examined two central questions about Japanese SIEs’ transnational career experiences: whether they achieve higher earnings growth than domestic workers, and whether returning to Japan penalizes their careers. Panel regression analyses provided strong support for the first hypothesis while challenging the second. Japanese expatriates experienced approximately 6% annual earnings growth after controlling for observable and unobservable factors, whereas domestic workers’ earnings remained virtually stagnant (around 1% annual growth). This substantial difference persisted across multiple model specifications and robustness checks, demonstrating that transnational career mobility yielded significant economic advantages over domestic career trajectories during Japan’s prolonged wage stagnation.

These findings challenge the dominance of methodological nationalism in social stratification research. Conventional mobility studies assume that career advancement occurs primarily within bounded national labor markets through organizational promotion systems. However, for Japanese middle-class workers facing three decades of domestic wage stagnation, transnational career experiences provided an alternative pathway to economic advancement that domestic career channels could not offer. This pattern reveals how transnational labor markets can create new opportunity structures when domestic mobility is constrained—a dynamic overlooked by frameworks that treat national labor markets as self-contained systems. The findings thus contribute to debates on how globalization reshapes career stratification processes beyond traditional national boundaries.

The second hypothesis—that returning to Japan penalizes SIEs’ earnings—received limited support. While descriptive results suggested lower earnings for returnees in Japanese-owned companies, multivariate analyses revealed that this apparent penalty largely reflected job characteristics and pre-migration conditions rather than return migration per se. Once job attributes, occupational positions, and working hours were controlled, the return effect became statistically insignificant. This finding challenges simplistic assumptions about return penalties and suggests that career outcomes depend more on the types of positions returnees secure than on the act of returning itself. This interpretation helps explain regional differences in return premiums documented in previous research: the contrasting patterns between European studies documenting positive premiums (Barrett and O’Connell, 2001; Barrett and Goggin, 2010) and ambiguous East Asian findings (Hao et al., 2017; Choi and Park, 2025) may reflect differences in how returnees are positioned within organizational structures rather than inherent regional variations in valuing international experience.

An important finding emerged regarding heterogeneity among Japanese expatriates. While earnings levels varied significantly—SIEs in multinational companies earned substantially more than those in Japanese-owned companies—earnings growth rates were similar across categories. This pattern suggests different mechanisms underlying transnational career advantages. SIEs in Japanese companies may benefit from the “Japanese premium” in regional supply chains, where Japanese language and cultural knowledge command value (Ishida et al., 2019), but remain subject to organizational hierarchies that limit their earnings levels relative to corporate expatriates. In contrast, SIEs in multinational companies likely leverage professional skills valued in global labor markets, achieving both higher earnings levels and comparable growth rates. These divergent patterns reveal how organizational contexts create differentiated opportunity structures even for migrants from the same origin country, highlighting the importance of examining within-group heterogeneity in transnational career research.

To be sure, the present study has several research limitations. The most critical issue is the sampling bias in the ADIOS-J sample, as it was not drawn using a conventional random sampling procedure. Unless a representative sampling frame is available, relying on non-random sampling eventually becomes the second-best option. Measuring job earnings for the ADIOS-J surveys also has room for improvement, as the ten-point scale is somewhat imprecise. Other outcomes, such as class categories, are to be investigated in future research by rigorously considering the compatibility of occupational statuses across countries.

In relation to the data issues, I acknowledge that this timeframe is limited given the 30-year context of Japan’s economic stagnation that motivated this study. The 2020–2022 period represents only a snapshot of what may be longer-term career mobility patterns. Despite that limitation, a similar wage trajectory result is observed in Japan over a more extended period; around 60% of wage earners in Japan experience wage growth rates from 0 to 0.05 (Arita, 2020), which are still lower than those of the Japanese SIEs. Accordingly, the JLPS sample results in the present study still seem to reflect Japan’s long economic stagnation, and the monetary incentive for working overseas appears to be increasing.

In addition to the data limitations, the analytical framework can be elaborated in future research. For example, because the present study primarily focuses on the Japanese SIEs’ transnational career achievement, including their return migration, the focal reference group is those who remain in the Japanese labor market (JLPS sample). Accordingly, comparisons between the Japanese SIEs and local workers are intentionally excluded. Meanwhile, from the immigration study perspective, it is imperative to compare the Japanese migrants with the majority in the host societies. Furthermore, the contexts of the receiving societies should be considered. To do so, it would be helpful to accumulate research on individual countries that are popular for Japanese migrants.

Nevertheless, using the rough outcome might still be reasonable for understanding the overall trends and differences among expatriates and domestic workers, as long as we do not intend to measure the exact income level. Addressing these issues in subsequent research leads us to rigorously test the validity of the current results.

## 7 Conclusion

This study makes three primary contributions to research on transnational career mobility and social stratification. First, methodologically, it attempts to better address the selection bias problem inherent in return migration research by utilizing longitudinal data that tracks both movers (those who stay abroad and those who return) and stayers (domestic workers). While acknowledging sampling limitations, this design allows for more rigorous examination of return migration effects than cross-sectional comparisons that conflate selection into return with return outcomes. Second, empirically, it provides systematic evidence on middle-class migrants’ earnings trajectories from a high-income country—a population largely overlooked in existing research that predominantly focuses on migrants from middle- and low-income countries. The findings reveal that Japanese SIEs experienced substantial earnings growth (approximately 6% annually) while domestic workers faced near-stagnant wages, demonstrating that transnational career mobility can serve as an alternative pathway to economic advancement when domestic channels are constrained. Third, theoretically, by challenging methodological nationalism’s assumption that career mobility occurs within bounded national contexts, this study demonstrates how transnational labor markets create new stratification dynamics even for workers from traditionally advantaged origin countries.

The findings have important implications for understanding the mechanisms underlying transnational career advantages. The study reveals that earnings growth advantages for Japanese SIEs were sustained regardless of organizational context, yet earnings levels varied substantially between those in Japanese-owned versus multinational companies. This pattern suggests that different forms of human and social capital operate in transnational labor markets: the “Japanese premium” based on language and cultural knowledge in regional supply chains versus professional skills valued in global labor markets. Critically, the study also reconceptualizes return penalties: the apparent economic costs of returning to Japan largely reflected job characteristics and pre-migration conditions rather than return migration itself. This finding suggests that institutional contexts shape not whether international experience is valued, but rather how returnees navigate job matching and organizational integration processes.

These findings have broader implications for understanding middle-class migration in an era of economic globalization and regional integration. As domestic labor market opportunities become increasingly constrained in some high-income countries—exemplified by Japan’s three-decade wage stagnation—transnational career mobility may become an increasingly important alternative to domestic career advancement for middle-class workers. However, the sustainability of these advantages depends on the transnational labor market structure—particularly the extent to which origin-country-specific premiums (such as Japanese-ness) persist as organizations globalize and adopt more standardized management practices. Future research should examine how organizational contexts beyond national ownership (such as industry sectors and firm strategies) shape transnational career opportunities, compare Japanese SIEs’ outcomes with local workers in host societies to better understand their relative positioning, and investigate longer-term career trajectories to assess whether the earnings advantages observed in this study persist or converge over time. Understanding these dynamics is crucial as transnational career mobility becomes a more common feature of middle-class employment in an increasingly interconnected global economy.

## Data Availability

The datasets presented in this article are not readily available because they are currently under dissemination processes in a data repository. Requests to access the datasets should be directed to Kenji Ishida (ishidak@iss.u-tokyo.ac.jp).

## Footnotes

1 The relevant figures are from “Annual Report of Statistics on Japanese Nationals Overseas,” reported by the Ministry of Foreign Affairs (Accessed on the 5th of August 2025).

2 Prior study centers on the Japanese SIE workers in Japanese companies overseas. However, though it is still speculation, the Japanese language premium can be expected for the SIEs employed in non-Japanese multinational companies, which attempt to reach out their businesses in the Japanese market.

3 In this study, the ADIOS-J sample is assumed to consist of SIEs and CEs. Meanwhile, there may be some cases where CEs’ spouses work in the host countries under certain conditions. Accordingly, the analytical sample cannot always be considered SIEs and CEs in the theoretical sense. Such cases carry the risk of underestimating wage growth rates. However, as shown in the analysis results discussed below, the wage growth rate of the ADIOS-J sample is positively significant and larger than that of domestic workers in Japan. Therefore, while acknowledging the limitations of the data, it appears to be plausible to assume that the results of this study have a certain degree of validity.

4 The geographic distribution of the obtained sample does not correspond to the true target population, as it is contingent on the geographic distribution of registrants on these platforms. At the time of the Wave1 survey, the respondents’ regions of residence were as follows: Mainland China accounted for 8% of the total, Hong Kong 4%, Taiwan/South Korea 5%, Singapore 8%, Thailand 15%, Other Southeast Asia 10%, Western Europe and North America 47%, and Other Regions 3%. In the subsequent analysis, a model incorporating a dummy variable for regions other than Japan was also estimated; however, the results remained unaltered.

5 The “plm” package (ver. 2.6.4) in R (ver. 4.4.0) is used to perform the random and fixed effects models.

6 To perform multiple imputations in the present study, I utilized “Amelia (ver. 1.8.3)” and “mitools (ver. 2.4)” in R (ver. 4.4.0).
